# The Relationship Between Famine Exposure During Early Life and Left Ventricular Hypertrophy in Adulthood

**DOI:** 10.3389/fnut.2022.898932

**Published:** 2022-05-31

**Authors:** Yu-qin Yan, Lin Liu, Shuo Sun, Ying-qing Feng, Jie Li, Yu-qing Huang

**Affiliations:** ^1^Department of Cardiology, People’s Hospital of Shenzhen Baoan District, Shenzhen, China; ^2^Department of Cardiology, Guangdong Provincial People’s Hospital, Guangdong Academy of Medical Sciences, Guangzhou, China

**Keywords:** left ventricular hypertrophy (LVH), famine exposure, early life, adulthood, famine

## Abstract

**Background:**

Although the evidence was still limited, some studies suggested that childhood malnutrition might affect cardiac function and structure in adulthood. To address the knowledge gap, this study investigated if the Great Chinese Famine exposure during early life had affected left ventricular hypertrophy (LVH).

**Methods:**

This research was a cross-sectional study. It included participants who had cardiac ultrasound assessments and were born in Guangdong, China, from 1 October 1952 to 30 September 1964. They were classified according to their exposure period to famine, namely, no exposure, fetal-, early-, mid-, and late childhood. Multivariate logistic regression and subgroup analysis have been conducted to determine the odds ratio (*OR*) and confidence intervals (*CI*s) between famine exposure and LVH.

**Results:**

This research included 2,543 participants, 1,612 women, their mean age was 59.07 ± 3.65 years, and 704 participants had LVH. LVH prevalence was 122 (23.6%), 87 (25.1%), 133 (27.3%), 184 (29.2%), and 178 (31.7%), in non-, fetal-, early-, mid-, and late-childhood exposed groups, respectively (*p* = 0.031), while in the non-exposed group, the *OR*s for developing carotid plaque as a result of fetal, early-, mid- to late-childhood exposure were 1.08 (95% *CI*: 0.76, 1.59, *p* = 0.619), 1.24 (95% *CI*: 1.03, 1.79, *p* = 0.031), 1.49 (95% *CI*: 1.10, 2.01, *p* = 0.009), and 1.64 (95% *CI*: 1.25, 2.18, *p* = 0.001), respectively (*p* for trend = 0.003). There was no interactive effect between gender, obesity, or hypertension history with how the famine influenced LVH, as the subgroups analyses demonstrated (all *p* for interaction > 0.05).

**Conclusion:**

This research has demonstrated the potential relationship between Great Chinese Famine exposure during childhood and LVH in adults.

## Introduction

Left ventricular growth, also known as left ventricular hypertrophy (LVH), occurs as a result of growth in the size of cardiomyocytes due to the coexistence of hemodynamic and non-hemodynamic components (1, 2). It was generally accepted that LVH was a common problem and could occur due to many disorders, such as hypertension, hypertrophic cardiomyopathy, aortic stenosis, infiltrative heart muscle disease, metabolic disorders, athletic training, and storage (3). Early detection and prevention of LVH were necessary because progressive LVH could lead to maladaptation and develop into progressive left ventricular dysfunction or heart failure, and seriously threaten the patient’s life (3). LVH is currently believed to be the result of genetics and the environment, and its pathogenesis has not been fully elucidated. Recently, the relationship between nutritional status and cardiovascular diseases (CVDs) has gained increasing interest, especially nutritional status during early life. More importantly, previous disease hypotheses suggested that the risk of developing the disease in adulthood was closely related to nutritional and environmental factors during the fetal or early life childhood stage (4, 5). This theory may explain why most chronic CVDs, such as coronary heart disease and hypertension, were associated with the Great Chinese Famine exposure in childhood in the Netherlands, Ukraine, and Great Chinese Famine (6–9). Nevertheless, until today, this association was still pending for verification in multiple populations.

## Materials and Methods

### Study Population

**FIGURE 1:**
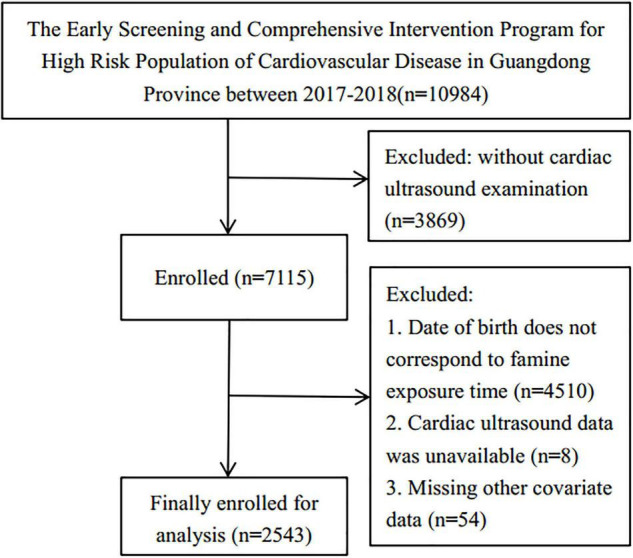
Research flowchart.

The data for this study were retrieved from The Early Screening and Comprehensive Intervention Program for High CVD Risk Population, which included participants born in Guangdong, China. The program was within the China-PEACE Million Persons Project, funded by the government to identify the at-risk population who may develop CVD in China (10, 11). The cardiac ultrasound assessment was conducted on 10,984 persons in Guangdong from 01/01/2017 to 31/12/2018. This research included participants who had this assessment and were born from 01/10/1952 to 30/9/1964, while participants born from 01/10/1958 to 30/09/1959 and 01/10/1961 to 30/09/1962 were eliminated due to the uncertain start and end dates for the Chinese famine’s start and end dates. In addition, participants who lacked data on covariates were excluded. Consequently, 2,543 participants were enlisted for the analysis (Figure 1). The Institute of Guangdong Provincial People’s Hospital’s ethics committee has approved this research protocol [No.GDREC2016438H (R2)].

### Famine Exposure

From 1959 to 1961, the Great Chinese Famine occurred. The participants were classified into five groups (12, 13) as follows, (1) non-, (2) fetal-, (3) early-, (4) mid-, and (5) late-childhood exposed groups, which represents participants born between 01/10/1962 and 30/09/1964 (*n* = 517), 01/10/1959 and 30/09/1961 (*n* = 346), 01/10/1956 and 30/09/1958 (*n* = 488), 01/10/1954 and 30/09/1956 (*n* = 630), and 01/10/1952 and 30/09/1954 (*n* = 562), respectively.

### Left Ventricular Hypertrophy Measurement

All examinations were conducted using a standardized approach by the same sonographer. The cardiac ultrasound assessment results were collected by the Vivid-S6 and 2.5–3.5 MHz-phased array probe usage, such as two-dimensional, M-mode, and Doppler ultrasound (12, 13). The left ventricular dimension, posterior wall thickness, inter-ventricular septum, left ventricular septum, and left ventricular end-diastolic diameter were measured using the parasternal long-axis view’s procedures following the Echocardiography American Society guidelines (14). The left ventricular mass index (LVMI) and left ventricular mass (LVM) counting were performed using the Devereux formula (15). The LVH was graded as LVMI >115 g/m^2^ and >95 g/m^2^ in men and women, respectively (16).

### Covariate Data Collection

Face-to-face interviews were conducted to collect socio-demographic data, such as gender, age, education, income and residential area, lifestyle behaviors, such as smoking and drinking, as well as chronic diseases (hypertension, coronary heart disease, diabetes, and stroke), and current medications (hypoglycemic, antihypertensive, and lipid-lowering drugs). Besides, height, weight, blood pressure, blood glucose, triglyceride, low- and high-density lipoprotein, and total cholesterol were also measured. The body mass index (BMI) was calculated, and BMI ≥25 kg/m^2^ was considered overweight (17). Participants who reported having diabetes, using glucose-lowering drugs, or having a fasting blood glucose level ≥126 mg/dl were classified as diabetic (18), while those who reported having hypertension, using antihypertensive drugs, or having a blood pressure ≥140/90 mmHg were classified as hypertensive (16).

### Statistical Analysis

The continuous and categorical variables were reported as mean ± standard deviation (SD), and a frequency or percentage, respectively. A normality test was first performed on all continuous variable data; comparisons that passed the normality test were analyzed by a Student *t*-test, and those that did not were analyzed with the Mann–Whitney *U*-test. The chi-square test was used for categorical variables to compare the baseline categorical characteristics. Multiple group comparisons passing the normality test were analyzed using analysis of variance (ANOVA) with *post hoc* tests, whereas non-parametric multiple group comparisons were analyzed using the Kruskal–Wallis test with Dunnett’s *post hoc* testing when ANOVA assumptions were not met. Categorical characteristics were compared using the chi-square test, and the Bonferroni method was applied to correct the value of *p* when comparing the multiple groups in pairs. For evaluating famine exposure and LVH association, the crude and adjusted odds ratio (*OR*) values were calculated. For determining the *OR* and confidence interval (*CI*), the multivariate logistic regression and interaction test had been used. No covariate was adjusted in model I, while age and gender were adjusted in model II. Meanwhile, age, gender, region, education, income, smoking, drinking, BMI, low-density lipoprotein cholesterol (LDL-C), hypertension, diabetes, stroke, coronary heart disease, and lipid-lowering drugs were adjusted in model III. Subgroups and interaction analyses were performed based on gender, BMI (≥25.0 or <25 kg/m^2^), and hypertension history. A two-sided *p* <0.05 was considered statistically significant. R version 3.3.2 had been used to conduct all the statistical analyses.

## Results

### Participants’ Characteristics

This research has included 2,543 participants, of which 1,612 were women, whose mean age was 59.07 ± 3.65 years. As demonstrated in Table 1, compared with subjects without LVH, subjects with LVH had older age, higher blood pressure, higher BMI, higher levels of LVM, LVMI, posterior wall (PW), inter-ventricular septum (IVS), and left ventricular end diastolic diameter (LVEDD), a higher proportion of women, lower proportion of participants living in urban areas, lower education level, lower smoking and drinking rate, accompanied with a higher prevalence of hypertension and the use of antihypertensive drugs (all *p* < 0.05). Table 2 shows that the LVH prevalence in non-, fetal-, early-, mid-, and late-childhood exposed groups was 122 (23.6%), 87 (25.1%), 133 (27.3%), 184 (29.2%), and 178 (31.7%), respectively (*p* = 0.031). Significant subgroup differences were noticed in age, systolic blood pressure, LVMI, education level, history of hypertension, and taking antihypertensive drugs (all *p* < 0.05).

**TABLE 1:** Baseline characteristics between subjects with and without LVH group.

	Overall	No-LVH	LVH	*P*
Number	2,543	1,839	704	
Age, years	59.07 ± 3.65	58.92 ± 3.68	59.44 ± 3.52	0.001
SBP, mmHg	143.44 ± 22.99	141.02 ± 22.64	149.77 ± 22.72	< 0.001
DBP, mmHg	83.17 ± 12.38	82.34 ± 12.14	85.36 ± 12.76	< 0.001
BMI, kg/m^2^	24.79 ± 3.35	24.51 ± 3.30	25.52 ± 3.36	< 0.001
TC, mmol/L	5.84 ± 1.52	5.85 ± 1.54	5.81 ± 1.48	0.459
TG, mmol/L	1.93 ± 1.08	1.91 ± 1.04	2.00 ± 1.16	0.065
HDL-C, mmol/L	1.64 ± 0.47	1.63 ± 0.47	1.66 ± 0.46	0.103
LDL-C, mmol/L	3.40 ± 1.26	3.43 ± 1.27	3.29 ± 1.21	0.017
FBG, mmol/L	6.14 ± 1.90	6.14 ± 1.94	6.16 ± 1.78	0.786
LVM, g	150.07 ± 39.48	135.67 ± 28.08	187.68 ± 40.21	< 0.001
LVMI, g/m^2^	91.85 ± 21.09	82.75 ± 13.03	115.63 ± 19.54	< 0.001
PW, mm	9.50 ± 1.52	9.13 ± 1.21	10.47 ± 1.80	< 0.001
IVS, mm	9.77 ± 1.41	9.37 ± 1.14	10.82 ± 1.48	< 0.001
LVEDD, mm	45.32 ± 4.34	44.26 ± 3.87	48.09 ± 4.27	< 0.001
Gender—female (%)	1,612 (63.4)	1,058 (57.5)	554 (78.7)	< 0.001
Urban (%)	766 (30.1)	591 (32.1)	175 (24.9)	< 0.001
Hypertension (%)	1,565 (61.5)	1,047 (56.9)	518 (73.6)	< 0.001
Diabetes (%)	502 (19.7)	348 (18.9)	154 (21.9)	0.094
Stroke (%)	36 (1.4)	31 (1.7)	5 (0.7)	0.062
CHD (%)	93 (3.7)	72 (3.9)	21 (3.0)	0.262
Education-High school or above (%)	576 (22.7)	457 (24.9)	119 (16.9)	< 0.001
Income-more than 50 k per years (%)	1,282 (50.4)	953 (51.8)	329 (46.7)	0.022
Current smoke (%)	475 (18.7)	405 (22.0)	70 (9.9)	< 0.001
Current drinking (%)	134 (5.3)	114 (6.2)	20 (2.8)	0.001
Lipid-lowering drug (%)	155 (6.1)	99 (5.4)	56 (8.0)	0.015
Antihypertensive drug (%)	764 (30.0)	480 (26.1)	284 (40.3)	< 0.001
Hypoglycemic drug (%)	206 (8.1)	145 (7.9)	61 (8.7)	0.519

*Continuous variables were expressed as the mean ± standard deviation (SD), and categorical variables were described as frequencies and percentages.*

*A normality test was first performed on all continuous variable data; comparisons that passed the normality test were analyzed by a Students t-test, and those that did not were analyzed with the Mann–Whitney U-test.*

*For comparing the baseline categorical characteristics, the chi-square test was used for categorical variables.*

*LVH left ventricular hypertrophy, LVM left ventricular mass, LVMI left ventricular mass index, IVS inter-ventricular septum, PW posterior wall, LVEDD left ventricular end diastolic diameter, CHD coronary heart disease, SBP systolic blood pressure, DBP diastolic blood pressure, FBG fasting blood glucose, TC total cholesterol, TG triglyceride, LDL-C low-density lipoprotein cholesterol, HDL-C high-density lipoprotein cholesterol, BMI body mass index.*

**TABLE 2:** Baseline characteristics among different famine exposure groups.

	No exposure	Fetal exposure	Early childhood	Mid childhood	Late childhood	*P*
Number	517	346	488	630	562	
Age, years	53.32 ± 0.67	56.34 ± 0.66*	59.34 ± 0.64*^†^	61.33 ± 0.67*^†‡^	63.25 ± 0.62*^†‡^§	< 0.001
SBP, mmHg	140.19 ± 23.45	140.77 ± 22.30	143.77 ± 22.64*^†^	144.10 ± 22.22*^†^	147.05 ± 23.60*^†‡^§	< 0.001
DBP, mmHg	84.25 ± 13.00	83.34 ± 13.20	83.13 ± 12.46	82.30 ± 11.75	83.08 ± 11.87	0.128
BMI, kg/m^2^	25.10 ± 3.40	25.05 ± 3.39	24.71 ± 3.47*^†^	24.41 ± 3.19*^†^	24.86 ± 3.32*^†^	0.004
TC, mmol/L	5.95 ± 1.47	5.96 ± 1.55	5.82 ± 1.53	5.81 ± 1.52	5.72 ± 1.53*^†^	0.062
TG, mmol/L	1.97 ± 1.10	1.92 ± 1.14	1.91 ± 1.04	1.88 ± 1.04	1.99 ± 1.09	0.397
HDL-C, mmol/L	1.63 ± 0.48	1.67 ± 0.48	1.66 ± 0.47	1.64 ± 0.45	1.61 ± 0.46	0.358
LDL-C, mmol/L	3.53 ± 1.29	3.48 ± 1.25	3.35 ± 1.23*	3.40 ± 1.25*	3.26 ± 1.26*^†‡^§	0.008
FBG, mmol/L	6.09 ± 1.94	6.01 ± 1.61	6.15 ± 1.89^†^	6.06 ± 1.64^‡^	6.36 ± 2.25*^†‡^§	0.024
LVM, g	148.66 ± 39.72	145.82 ± 35.18*	148.59 ± 40.79^†^	150.74 ± 38.22*^†^	154.51 ± 41.65*^†‡^§	0.013
LVMI, g/m^2^	89.49 ± 20.55	89.30 ± 18.93	91.44 ± 21.24*^†^	93.00 ± 20.37*^†‡^	94.68 ± 23.04*^†‡^§	< 0.001
PW, mm	9.43 ± 1.19	9.34 ± 1.12	9.39 ± 1.28	9.66 ± 2.20*^†‡^	9.59 ± 1.22*^†‡^	0.002
IVS, mm	9.70 ± 1.47	9.70 ± 1.37	9.76 ± 1.48	9.78 ± 1.35	9.89 ± 1.37	0.177
LVEDD, mm	45.30 ± 4.45	45.06 ± 3.80	45.22 ± 4.32	45.22 ± 4.45	45.71 ± 4.40	0.174
Gender—female (%)	184 (35.6)	108 (31.2)*	168 (34.4)	246 (39.0)*^†‡^	225 (40.0)*^†‡^§	0.040
Urban (%)	136 (26.3)	119 (34.4)*	142 (29.1)*^†^	202 (32.1)*^‡^	167 (29.7)*^†^	0.092
Hypertension (%)	278 (53.8)	191 (55.2)*	306 (62.7)*^†^	397 (63.0)*^†^	393 (69.9)*^†‡^§	< 0.001
Diabetes (%)	100 (19.3)	61 (17.6)	106 (21.7)^†^	111 (17.6)^‡^	124 (22.1)^†^	0.204
Stroke (%)	6 (1.2)	3 (0.9)	5 (1.0)	15 (2.4)*^‡^	7 (1.2) §	0.211
CHD (%)	14 (2.7)	12 (3.5)	21 (4.3)	28 (4.4)	18 (3.2)	0.497
Education-High school or above (%)	119 (23.0)	100 (28.9)*	133 (27.3)*^†^	118 (18.7)*^†‡^	106 (18.9)*^†‡^	< 0.001
Income-more than 50 k per years (%)	266 (51.5)	180 (52.0)	242 (49.6)	330 (52.4)	264 (47.0)*^†^§	0.361
Current smoke (%)	99 (19.1)	57 (16.5)*	79 (16.2)*	137 (21.7)^†‡^	103 (18.3)	0.130
Current drinking (%)	29 (5.6)	19 (5.5)	29 (5.9)	32 (5.1)	25 (4.4)	0.844
Lipid-lowering drug (%)	16 (3.1)	18 (5.2)*	27 (5.5)*	45 (7.1)*^†‡^	49 (8.7)*^†‡^§	0.002
Antihypertensive drug (%)	108 (20.9)	85 (24.6)*	154 (31.6)*^†^	205 (32.5)*^†^	212 (37.7)*^†‡^§	< 0.001
Hypoglycemic drug (%)	31 (6.0)	24 (6.9)	41 (8.4)*^†^	48 (7.6)*	62 (11.0)*^†‡^§	0.034
LVH (%)	122 (23.6)	87 (25.1)	133 (27.3)*^†^	184 (29.2)*^†‡^	178 (31.7)*^†‡^§	0.031

*Continuous variables were expressed as the mean ± SD, and categorical variables were described as frequencies and percentages.*

*Multiple group comparisons passing normality test were analyzed using analysis of variance (ANOVA) with post hoc tests, whereas non-parametric multiple group comparisons were analyzed using the Kruskal–Wallis test with Dunnett’s post hoc testing, when ANOVA assumptions were not met.*

*Categorical characteristics were compared using the chi-square test, and the Bonferroni method was applied to correct the value of p when comparing the multiple groups in pairs.*

*p < 0.05 compared with the no exposure group.

*^†^p < 0.05 compared with the fetal exposure group.*

*^‡^p < 0.05 compared with the early childhood group.*

*^§^p < 0.05 compared with the mid childhood group.*

*LVH left ventricular hypertrophy, LVM left ventricular mass, LVMI left ventricular mass index, IVS inter-ventricular septum, PW posterior wall, LVEDD left ventricular end diastolic diameter, CHD coronary heart disease, SBP systolic blood pressure, DBP diastolic blood pressure, FBG fasting blood glucose, TC total cholesterol, TG triglyceride, LDL-C low-density lipoprotein cholesterol, HDL-C high-density lipoprotein cholesterol, BMI body mass index.*

### Famine Exposure and LVH Associations

Table 3 shows the famine exposure and LVH association as explored by multivariate logistic regression analysis. In model I, with no variables adjusted, the *OR*s for LVH from fetal-, early-, mid-, and late-childhood exposure were 1.09 (95% *CI*: 0.79, 1.49, *p* = 0.603), 1.21 (95% *CI*: 0.91, 1.61, *p* = 0.183), 1.34 (95% *CI*: 1.02, 1.75, *p* = 0.033), and 1.50 (95% *CI*: 1.15, 1.97, *p* = 0.003) (*p* for trend was 0.001), respectively. In model II, age and gender were adjusted, the *OR*s for LVH from fetal-, early-, mid-, and late-childhood exposure were 1.05 (95% *CI*: 0.76, 1.45, *p* = 0.779), 1.23 (95% *CI*: 0.93, 1.68, *p* = 0.134), 1.49 (95% *CI*: 1.14, 1.97, *p* = 0.004), and 1.63 (95% *CI*: 1.24, 2.09, *p* = 0.002) (*p* for trend was < 0.001), respectively. In model III, age, gender, region, education, income, smoking, drinking, BMI, LDL-C, hypertension, diabetes, stroke, coronary heart disease, and lipid-lowering drugs were all adjusted, in comparison with the non-exposed group, the *OR*s for developing LVH as a result of fetal, early-, mid-, and late-childhood exposure were 1.08 (95% *CI*: 0.76, 1.59, *p* = 0.619), 1.24 (95% *CI*: 1.03, 1.79, *p* = 0.031), 1.49 (95% *CI*: 1.10, 2.01, *p* = 0.009), and 1.64 (95% *CI*: 1.25, 2.18, *p* = 0.001), respectively (*p* for trend = 0.003).

**TABLE 3:** Relationship between famine exposure and LVH among different groups.

	Model I			Model II			Model III		
	OR	95% CI	*P*	OR	95% CI	*P*	OR	95% CI	*P*
No exposure	Ref			Ref			Ref		
Fetal exposure	1.09	0.79, 1.49	0.603	1.05	0.76, 1.45	0.779	1.08	0.67, 1.59	0.619
Early childhood exposure	1.21	0.91, 1.61	0.183	1.23	0.93, 1.68	0.134	1.24	1.03, 1.79	0.031
Mid childhood exposure	1.34	1.02, 1.75	0.033	1.49	1.14, 1.97	0.004	1.49	1.10, 2.01	0.009
Late childhood exposure	1.50	1.15, 1.97	0.003	1.63	1.24,2.09	0.002	1.64	1.25, 2.18	0.001
P for trend			0.001			< 0.001		0.003	0.003

*Data are presented as OR and 95% CI. Values of p are for the comparison of the difference in famine exposure groups.*

*OR odds ratio, CI confidence interval, LVH left ventricular hypertrophy.*

*Model I adjust for none;*

*Model II adjust for age and gender;*

*Model III adjust for age, gender, region, education, income, smoking, drinking, body mass index, LDL-C, hypertension, diabetes, stroke, coronary heart disease, and lipid-lowering drugs.*

### Subgroup Analysis

Table 3 demonstrates that subgroup analyses and interaction tests were performed according to gender, BMI, and history of hypertension. As shown in Table 4, we found that famine exposure in early-, mid-, and late childhood was linked to high risks for LVH in women, subjects without hypertension, and subjects with BMI < 25 kg/m^2^. In addition, no famine exposure in the fetal period and LVH in adults’ correlation were found. Nevertheless, there were no famine exposure and LVH interaction in any subgroup variable (all *p* for interaction > 0.05).

**TABLE 4:** Subgroup analysis among different famine exposure groups.

Group	No exposure	Fetal exposure			Early childhood			Mid childhood			Late Childhood			*P* for interaction
		OR	95%CI	*P*	OR	95%CI	*P*	OR	95%CI	*P*	OR	95%CI	*P*	
Gender														0.140
Male (*n* = 931)	Ref	0.99	0.69, 1.49	0.571	1.04	0.72, 1.51	0.532	1.06	0.75, 1.97	0.415	1.18	0.63, 2.23	0.210	
Female (*n* = 1612)	Ref	1.14	0.60, 2.98	0.334	1.35	1.01, 2.41	0.040	1.68	1.19, 2.38	0.024	1.72	1.26, 2.10	0.003	
Hypertension														0.203
No (*n* = 978)	Ref	1.10	0.81, 2.06	0.316	1.31	1.05, 2.32	0.036	1.73	1.16, 2.98	0.028	1.84	1.28, 3.29	0.017	
Yes (*n* = 1565)	Ref	0.98	0.59, 1.29	0.675	0.99	0.66, 1.47	0.350	1.38	0.96, 1.81	0.087	1.30	0.97, 2.10	0.160	
BMI, kg/m^2^														0.941
≥ 0.9 (*n* = 1152)	Ref	1.02	0.63, 1.34	0.553	1.17	0.75, 1.82	0.476	1.33	0.88, 2.04	0.183	1.36	0.89, 2.09	0.164	
< 25 (*n* = 1391)	Ref	1.18	0.90, 2.00	0.328	1.34	1.05, 2.19	0.040	1.55	1.14, 2.43	0.008	1.76	1.33, 2.52	0.001	

*Data are presented as OR and 95% CI.*

*Values of p are for the comparison of the difference in subgroup condition.*

*OR odds ratio, CI confidence interval, BMI body mass index.*

## Discussion

In the present study, the Great Chinese Famine exposure during the early, middle, and late stages of childhood, was associated with LVH development in adulthood. Although the interaction tests were not significant, preliminary evidence might suggest that the influence of famine exposure on the LVH development in adulthood being more pronounced in women, subjects with normal weight, and people without hypertension.

Fetal development and infancy were early life stages defined by organ structure and systems’ rapid growth, development, and maturation (19). The food quality and quantity taken by pregnant women or infants may have long-lasting and profound impacts on growing tissues and it may alter the body’s response pattern (19–22). After multivariate adjustment, we demonstrated the association between famine exposure in childhood and LVH in adulthood. However, research about the Dutch famine demonstrated that the prenatal famine and adult LVH (estimated by electrocardiographic) had no significant association (23). This discrepancy in findings might be attributed to the variation in LVH assessment methods, ethnicity, the extent and timing of exposure to famine, and covariates being adjusted.

In the current research, we noticed that exposure to the fetal and early childhood had less effect on the development of LVH in adulthood than exposure to middle and late childhood. We speculated that individuals who were extremely malnourished in the fetus or early childhood may have been miscarried or died, and the surviving individuals received additional attention from their families. However, the precise mechanism of this phenomenon is yet to be explored.

In addition, subgroup analysis demonstrated a greater famine exposure effect during childhood on LVH in women than men. On the one hand, the traditional Chinese ideology of “prioritizing boys over girls” has played a major role. In traditional Chinese society, the family paid more attention to boys, so they may have received better nutrition as they grew up. On the other hand, most participating women in this study were already in perimenopause or menopause, and the estrogen’s protective effect on the cardiovascular system was no longer obvious. Additionally, we found that famine exposure in childhood had a greater effect on LVH in adults among people without comorbid hypertension, as well as in normal weight subjects. It is possible that famine exposure during childhood was strongly linked to hypertension development and obesity in adulthood (24, 25), and the possible nutritional status of non-hypertensive and obese individuals was deficient. However, it is notable that the interaction tests were not significant, so the proposed mechanisms as mentioned above should be verified in further studies.

Although famine exposure in childhood and LVH in adulthood had a strong association, the mechanism was not entirely clear. First, subjects who passed the famine alive may have catch-up growth and may result in over-nutrition, which has a significant association with cardiac structure and function (26, 27). Second, a previous study found that exposure to famine during gestation was linked to insulin resistance and increased oxidative stress responsiveness (8). In addition, poor nutrient status, the inflammation, and oxidative stress were closely related, which may have a vital impact on the immune system (28). Finally, nutritional status was closely related to endothelial dysfunction (29, 30) and sympathetic activity (31, 32). Numerous previous studies from basic science have confirmed that oxidative stress, inflammatory processes, endothelial dysfunction, and insulin resistance play important roles in developing LVH (33–36).

There were several strengths in the present study. On the one hand, this research was one of the first studies in indicating that famine exposure during early life could develop an LVH risk in adults. On the other hand, the present study provided new ideas for the early prevention of LVH. In addition, since the Great Chinese Famine had a huge impact on all of China, theoretically, our research findings can be extrapolated to other regions of China. Meanwhile, we should be aware of several limitations of this study. First, the cardiac ultrasound data acquisition was performed manually, so there may be measurement errors. Second, it cannot draw a causal relationship between famine exposure in childhood and LVH in adulthood given that it was a cross-sectional survey. Well-designed prospective studies were needed to clarify this association in the future. Third, some variables as measured at baseline, such as current use of medication and chronic diseases history, came from self-reported data and may have recall bias. Fourth, although the famine exposure was classified by the birthdate of the participants, the influence of age on LVH could not be fully eliminated, which was a common limitation for studies examining famine-disease relationship. Fifth, some populations may have died due to famine, which could have led to survivor bias on the influence of famine exposure and LVH development. Finally, the current study has no data on birth weight, time of pregnancy, and hematological or biological markers to assess nutritional status.

## Conclusion

In conclusion, the Great Chinese Famine exposure in childhood was linked to an increased LVH incidence in adults. No obvious association was observed for famine exposure in the fetal period and LVH, but exposure in early, middle, and late childhood were independently associated with LVH in adulthood. Our finding identifies the at-risk population that should receive more attention in preventing LVH.

## Data Availability Statement

The raw data supporting the conclusions of this article will be made available by the authors, without undue reservation.

## Ethics Statement

The studies involving human participants were reviewed and approved by the Ethics Committee at the Institute of Guangdong Provincial People’s Hospital [No. GDREC2016438H (R2)]. The patients/participants provided their written informed consent to participate in this study.

## Author Contributions

YY, YH, JL, and YF: conceptualization and study design. SS and LL: investigation. YY, JL, and YH: manuscript preparation. LL and YH: statistical analysis and data interpretation. All authors reviewed and approved this manuscript.

## Conflict of Interest

The authors declare that the research was conducted in the absence of any commercial or financial relationships that could be construed as a potential conflict of interest.

## Publisher’s Note

All claims expressed in this article are solely those of the authors and do not necessarily represent those of their affiliated organizations, or those of the publisher, the editors and the reviewers. Any product that may be evaluated in this article, or claim that may be made by its manufacturer, is not guaranteed or endorsed by the publisher.
